# Real-World Evidence of COVID-19 Patients’ Data Quality in the Electronic Health Records

**DOI:** 10.3390/healthcare9121648

**Published:** 2021-11-28

**Authors:** Samar Binkheder, Mohammed Ahmed Asiri, Khaled Waleed Altowayan, Turki Mohammed Alshehri, Mashhour Faleh Alzarie, Raniah N. Aldekhyyel, Ibrahim A. Almaghlouth, Jwaher A. Almulhem

**Affiliations:** 1 Medical Informatics and E-Learning Unit, Medical Education Department, College of Medicine, King Saud University, Riyadh 12372, Saudi Arabia; 436101646@student.ksu.edu.sa (M.A.A.); 436103134@student.ksu.edu.sa (K.W.A.); 436100746@student.ksu.edu.sa (T.M.A.); 434102757@student.ksu.edu.sa (M.F.A.); raldekhyyel@ksu.edu.sa (R.N.A.); Jalmulhem@ksu.edu.sa (J.A.A.); 2 Department of Medicine, College of Medicine, King Saud University, Riyadh 12372, Saudi Arabia; ialmaghlouth@ksu.edu.sa

**Keywords:** data quality, electronic health record, COVID-19, case identification, clinical documentation, medical informatics

## Abstract

Despite the importance of electronic health records data, less attention has been given to data quality. This study aimed to evaluate the quality of COVID-19 patients’ records and their readiness for secondary use. We conducted a retrospective chart review study of all COVID-19 inpatients in an academic healthcare hospital for the year 2020, which were identified using ICD-10 codes and case definition guidelines. COVID-19 signs and symptoms were higher in unstructured clinical notes than in structured coded data. COVID-19 cases were categorized as 218 (66.46%) “confirmed cases”, 10 (3.05%) “probable cases”, 9 (2.74%) “suspected cases”, and 91 (27.74%) “no sufficient evidence”. The identification of “probable cases” and “suspected cases” was more challenging than “confirmed cases” where laboratory confirmation was sufficient. The accuracy of the COVID-19 case identification was higher in laboratory tests than in ICD-10 codes. When validating using laboratory results, we found that ICD-10 codes were inaccurately assigned to 238 (72.56%) patients’ records. “No sufficient evidence” records might indicate inaccurate and incomplete EHR data. Data quality evaluation should be incorporated to ensure patient safety and data readiness for secondary use research and predictive analytics. We encourage educational and training efforts to motivate healthcare providers regarding the importance of accurate documentation at the point-of-care.

## 1. Introduction

The Electronic health record (EHR), primarily used for clinical care and billing purposes [1], has been arising as a potential source of patients’ data for clinical and translational research. In several applications, healthcare data can be used for secondary purposes [2,3,4,5] including deriving healthcare decisions, managing patients’ conditions, data exchange, building predictive models, and deriving new medical discoveries [1,6]. Researchers use EHR data due to the availability of big and real-time phenotypic data [1,7], less time for cohort construction, the availability of data for rare diseases, and cost-effectiveness [8].

The quality of EHR-based studies is highly reliant on the quality of EHR data. Data quality is “the ability of EHR-derived data to produce an accurate, reliable, and consistent aggregate-level picture of what is happening at the point-of-care” [9]. For secondary use of data to be used by researchers, it is vital to ensure that EHR data are high in quality [2,10], which improves the quality of care and organization overall performance [11], and ensures that accurate and valid conclusions are derived from the EHR. EHR users, “generators of data” and “consumers of data” [9], should understand EHR dataset limitations before its use by identifying sources of errors and recognizing the underline causes of errors [2,10]. Issues with the quality of data, such as incompleteness, inaccuracy, and inconsistency, can lead to threats to patient care and can result in risk consequences [11,12]. Inaccuracies in EHR data have been reported previously and can largely affect the quality of care and patient safety [11,12,13,14]. For example, Botsis et al. [13] found that only 1589 out of 3068 patients with ICD-9-CM diagnoses for pancreatic cancer had pathology reports documentations. Inconsistency is also observed when the same patient receives two different ICD9-CM codes for two types of diabetes (Type 1 ICD-9:250.01 and Type 2 ICD-9: 250.02). Inaccuracies can be also found in EHR, for instance, when the ICD-9 code for diabetes (250) is used rather than specifying if the diagnosis is Type 1 ICD-9:250.01 or Type 2 ICD-9: 250.02. These issues were usually originated at the point of care when a patient first encounters the medical facility. Several factors can contribute to low data quality, including human, managerial, and technical factors [11,15]. EHR data with low quality can “severely reduce the usability of data, mislead or bias the querying, analyzing and mining, and lead to huge loss” [16].

During the coronavirus disease (COVID-19) pandemic, EHRs were a crucial data source, as they provided essential information for clinicians and researchers in understanding the disease dynamics, treatment efficacy, and new investigations and interventions [17]. A high-quality EHR should be capable of identifying correct and accurate counts of COVID-19 positive cases, for example using their documented diagnoses information within EHR along with clinical findings, epidemiology, chest X-rays, and laboratory testing [9,18,19,20]. This information must be properly documented as generating high-quality EHR data for real-world applications and secondary use during crisis responses is challenging [9].

Despite the importance of the data and its quality during the COVID-19 pandemic, less attention has been given to data quality and limitations of EHR [21]. If inaccuracies are found in clinical data, it would cause a serious impact, especially during the COVID-19 pandemic where the public health response is guided by the research that highly depends on clinical data. Failures have been reported in defining COVID-19 cases accurately from EHRs, and there is a need to validate EHR data [22,23]. It is also reported that many EHR-based studies lacked transparency in EHR-driven phenotype identification [24]. Evaluating the quality of EHR records can be challenging and a level of manual review is needed to ensure high data quality and accuracy [24]. To advance the knowledge about COVID-19, the quality of EHR data needs to be assessed and issues need to be identified. With this, we identified the importance of evaluating the quality of COVID-19 data within EHR. This aimed to provide better patient safety, higher quality of care, and future applications of research and predictive models using machine learning and artificial intelligence (AI).

### Related Work

The current infrastructure and complexity of EHR systems vary across hospitals, which limits the capability of using EHR data for research purposes [9]. Data quality and related issues have been studied in many contexts, and the findings can vary across different institutions and different research studies [9,25,26,27,28]. Many such issues are generated during the documentation process at the point of care [28]. There can be various reasons for variability in performance across different institutions including social, cultural, and environmental aspects of a health information system [29]. For example, Santostefano et al. found that the documentation of the 10th version of International Classification of Diseases (ICD-10) code U07.1 was more common in symptomatic than asymptomatic patients [30]. ICD-10 codes are reasonably accurate for identifying COVID-19 patients as reported by Blatz et al. [25] (sensitivity = 90.5%, specificity = 99.9%) and Kadri et al. [31] (sensitivity = 98.01%, specificity = 99.04%). In contrast, ICD-10 codes are also known to give low sensitivity even though they are have high specificity [28]. Lynch et al. evaluated the performance of ICD-10 code U07.1 for identifying COVID-19 patients using a manual chart review as a gold standard, and they found that the performance was low [26]. Similarly, DeLozier et al. found that using laboratory testing (sensitivity = 93%) only to define COVID-19 patients outperformed the use of ICD-10 code U07.1 (sensitivity = 46.4%), which can be improved when combining the output of both definitions of ICD-10 and laboratory testing to yield a sensitivity of 100% [27]. Lynch et al. reported the use of ICD-10 codes either alone or supported with laboratory tests is not sufficient for surveillance and research [26], as ICD-10 codes do not appear to capture cases correctly [30]. In addition, the absence of a diagnostic code in the EHR does not necessarily represent the absence of the phenotype [28]. Furthermore, the results of cohort identification from EHR can vary even across different phenotypes, e.g., ICD-10 codes for congestive heart failure versus hypertension) [6].

There may be other data quality issues when utilizing EHR data in building registries and predictive models. DeLozier et al. developed a COVID-19 registry at a single academic medical center and found one-third of a COVID-19 cohort were missing demographic information and the lowest odds (OR 0.008) were in the positive individuals [27]. They also found the presence of false observations and the absence of true comorbidities. On the other hand, the performance of a machine-learning predictive model is highly reliant on the quality and accuracy of the training dataset and its outcome classes, i.e., patient outcomes [32]. Mamidi et al. developed a risk prediction model for COVID-19 utilizing a dataset composed of 7262 patients, of which 912 patients were diagnosed with COVID-19. The study showed that incorporating the correct ICD-10 codes help in deriving novel inferences of EHR data especially for medical symptoms and conditions that can increase the risk of COVID-19, such as cough, abnormalities of breath, chest pain, and allergic rhinitis. However, the accuracy of the ICD-10 code is still problematic in the classification task with up to 80% error rates [33].

EHR data might rarely be error-free; therefore, evaluating the quality of EHR data is important for deriving research-grade and computable phenotypes and public health real-time tracking and response [9,28,34]. The need for further studies in assessing data quality across different EHR systems has been reported by several studies [9,25,26,27,28]. Ann Marie Navar [35] provided an example of the COVID-19 data quality issue and stated that “the present example is one of many that show how far we remain from being able to use EHR data alone to conduct reliable, in-depth, and accurate observational research” [35]. Moreover, we found that most studies focused on only COVID-19 confirmed cases [25,26,27,28], one study focused on COVID-19 confirmed and susceptible cases [15], and none of these studies included COVID-19 probable cases [15,25,26,27,28]. Assessing the quality of symptom- and social-history-based definitions, such as COVID-19 susceptible and probable cases, is challenging and requires a manual chart review.

In this work, we aimed to evaluate the quality of COVID-19 patients’ data in the EHRs and their readiness for secondary use of data. The first objective is to compare the presence of documented COVID-19 signs and symptoms between structured diagnoses and problems lists and unstructured clinical notes. The second objective is to evaluate the accuracy of COVID-19 patients’ data in the EHR, and the challenges associated with its use.

## 2. Materials and Methods

### 2.1. Study Type

On 25 December 2020, we conducted a retrospective chart review to examine the documentation quality of COVID-19 patients’ records in structured and unstructured data.

### 2.2. Inclusion and Exclusion Criteria

We included all COVID-19 inpatient records documented during the year 2020. We excluded patients’ records with an admission date before 2020.

### 2.3. EHR System and Setting

The EHR system used at King Saud University Medical City (KSUMC) is Cerner PowerChart^®^ [36]. KSUMC is a tertiary care academic medical center, located in Riyadh Saudi Arabia. KSUMC has 10 multidisciplinary hospitals and centers with general and subspecialty medical services. KSUMC includes more than 1300 physicians, 853 residents and fellows, and around 2072 allied health personnel. KSUMC provides care to more than 1,229,628 outpatients and performs around 14,231 procedures yearly with a bed capacity of over 1200 [37]. Following the King Saud University Institutional Review Board (IRB) approval, we worked directly on data query and extraction from the EHR database with the Executive Department of Information Technology at KSUMC based on the description in the next section (Section 2.4).

### 2.4. Data Extraction and Chart Review

We identified COVID-19 inpatient records with final diagnoses using four main ICD-10 diagnosis codes shown in Table 1. The query extracted structured data from the EHR database including the medical record number (MRN), diagnosis code, diagnosis description, admission date and time, medical department, discharge disposition, and laboratory tests.

After extracting the structured data from the EHR database, four trained and authorized medical interns (M.A.A., K.W.A., T.M.A., M.F.A.) performed a manual chart review by directly accessing patient records stored in the EHR system. We developed a structured form (Table A1) according to the most recent COVID-19 case definitions published by the World Health Organization (WHO) [19] to collect the following: (1) Structured data: Clinical criteria symptoms within 10 days from diagnoses and problem lists, and (2) unstructured data: Clinical criteria symptoms within 10 days and epidemiological criteria from clinical notes, and chest imaging reports showing findings suggestive of COVID-19 disease.

### 2.5. COVID-19 Case Definition

**Figure 1:**
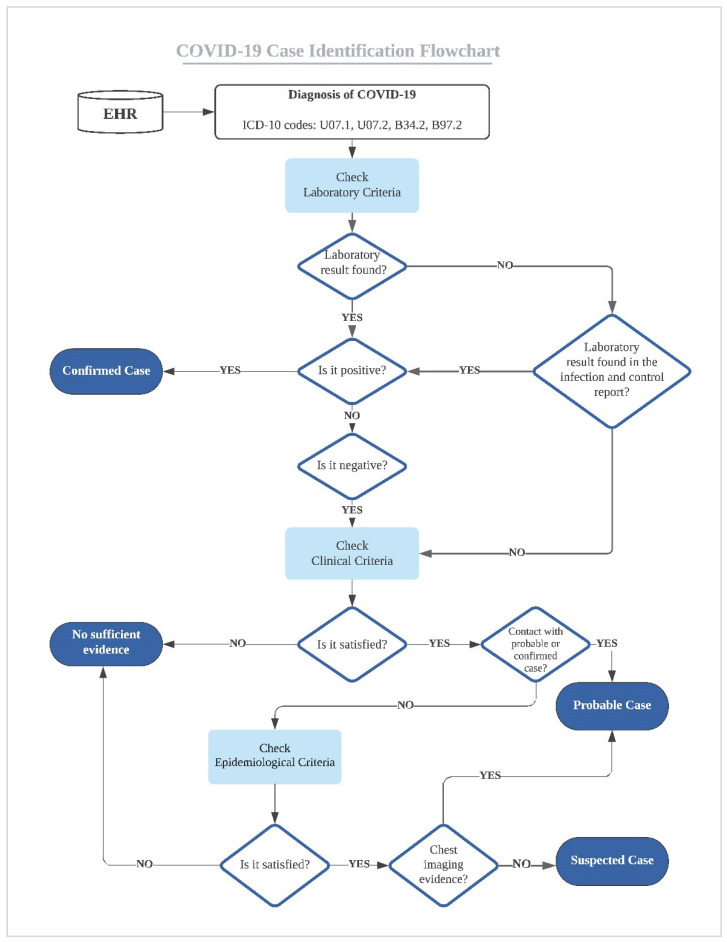
COVID-19 flowchart for case identification.

We followed the most recent COVID-19 case definitions guidance published by the WHO titled “Public health surveillance for COVID-19: interim guidance” [19]. The guidance includes four case definitions: (1) “confirmed case” is assigned when a patient satisfies the laboratory criteria positive for COVID-19 diagnosis; (2) “probable case” is assigned when a patient satisfies the clinical criteria and is in close contact with a confirmed or probable case of COVID-19 disease or suspected cases with diagnostic imaging evidence of COVID-19; (3) “suspected case” is assigned when a patient satisfies the clinical criteria and epidemiological criteria; and (4) “no sufficient evidence” is assigned if the presented data do not provide sufficient evidence to assign a diagnosis. We summarized the WHO’s case definition as a flowchart in Figure 1 and the descriptions for laboratory, clinical, and epidemiological criteria are in Table A2. Following the COVID-19 definition flowchart (Figure 1), we assigned cases in our study dataset. All assigned cases were validated by a second reviewer.

### 2.6. Data Quality Evaluation and Data Analysis

We applied the following two data quality measurements in our study: Inconsistently and inaccuracy. Inconsistency is defined as the information mismatch within the same EHR data source. The criterion for measuring inconsistency was assessed by identifying the data inconsistencies or disagreements between elements within the EHR [13]. Inaccuracy is defined as “non-specific, non-standards-based, inexact, incorrect, or imprecise information”, which can be “reflected as poor granularity of the diagnosis terms or disease classification codes and inadequate or non-standardized documentation of disease status” [13,38]. The criterion for measuring the inaccuracy was assessed by evaluating the documentation of the correct final diagnosis ICD-10 codes or the agreement with the general medical knowledge or information [13,38] (the WHO COVID-19 case definitions [19]).

We categorized the prevalence of COVID-19 symptoms based on the type of data, i.e., structured and unstructured clinical data. We used measures of diagnostic accuracy to evaluate the performance of ICD-10 codes and COVID-19 laboratory tests in identifying patients’ records with COVID-19 “confirmed cases”, which included [39].

(1) $$\mathrm{Specificity}\;=\frac{\mathrm{True}\;\mathrm{Positive}}{\mathrm{True}\;\mathrm{Positive}+\mathrm{False}\;\mathrm{Negative}},$$

(2) $$\mathrm{Specificity}\;=\frac{\mathrm{True}\;\mathrm{Negative}}{\mathrm{True}\;\mathrm{Negative}+\mathrm{False}\;\mathrm{Positive}},$$

(3) $$\mathrm{Accuracy}\;=\frac{\mathrm{True}\;\mathrm{Positive}+\mathrm{True}\;\mathrm{Negative}}{\mathrm{True}\;\mathrm{Positive}+\mathrm{False}\;\mathrm{Positive}+\mathrm{True}\;\mathrm{Negative}+\mathrm{False}\;\mathrm{Negative}}$$

Descriptive statistics of the COVID-19 dataset, COVID-19 signs and symptoms in structured and unstructured clinical data, and COVID-19 cases’ final interpretations based on COVID-19 case definition guidelines are presented in the results section. Data were analyzed and visualized using Microsoft Excel (version 2017, Microsoft Office 365) [40] and the statistical software R version 4.0.3 [41].

## 3. Results

We extracted and manually reviewed a total of 328 inpatient records. Admission dates in our dataset ranged from 17 March 2020 to 25 December 2020. The majority of the records represented male patients (*n* = 189, 57.62%), Saudi nationality (*n* = 233, 71.04%), and between 31 and 40 years old (*n* = 69, 21.04%). Within our dataset, the number of patients who died during hospitalization was (*n* = 28, 8.54%) with ages ranging from 12 to 90 years old and an average of 60 years. Patients in our dataset received care from 361 medical departments. All patient records reviewed in our study had complete descriptive data as indicated in Table 2.

**Figure 2:**
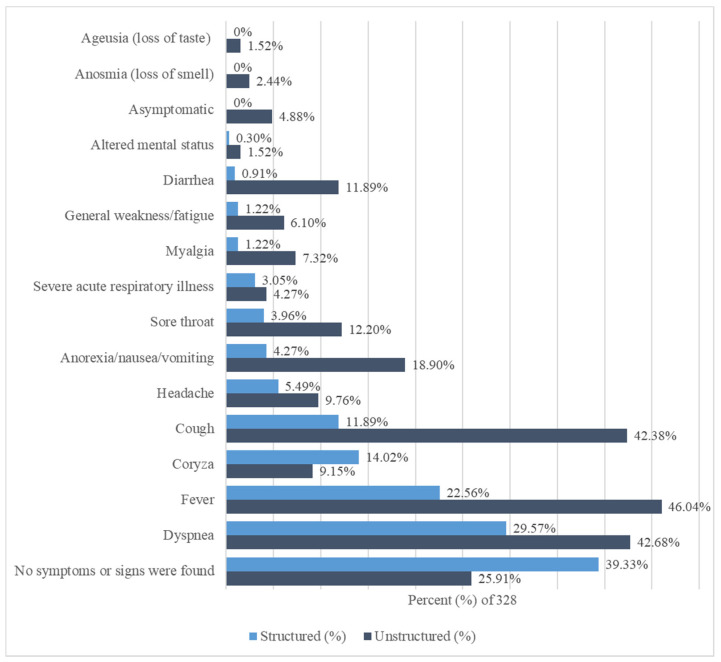
COVID-19 signs and symptoms in structured and unstructured clinical data. The signs and symptoms are sorted by structured data’s percentages from highest (down) to lowest (up).

We observed variations in the documentation and prevalence of reported signs and symptoms between structured and unstructured data (Figure 2 and Table A3). The total number of reviewed unstructured was 3348 notes, which were found in triage notes, nurse notes, ER notes, infection control notes, radiology reports, and consultant notes. Documentation of symptoms was higher in unstructured data (*n* = 725) than in structured data (*n* = 323). In structured data, the top five frequent symptoms were dyspnea (*n* = 97, 29.57%), fever (*n* = 74, 22.56%), coryza (*n* = 46, 14.02%), cough (*n* = 39, 11.89%), and headache (*n* = 18, 5.49%). However, symptoms of ageusia (loss of taste) and anosmia (loss of smell), as well as those asymptomatic were not reported in structured data. In unstructured data, the top five frequent symptoms were fever (*n* = 151, 46.04%), dyspnea (*n* = 140, 42.68%), cough (*n* = 139, 42.38%), anorexia/nausea/vomiting (*n* = 62, 18.90%), and sore throat (*n* = 40, 12.20%). There were no reported symptoms found in (*n* = 129, 39.33%) of structured diagnoses compared to (*n* = 85, 25.91%) found in unstructured clinical notes. Overall, the reporting of symptoms was higher in unstructured data than in structured data.

Table 3 shows results for cases identified linked to each diagnostic criteria (COVID-19 ICD-10 codes, COVID-19 laboratory test, history of contact with a probable or confirmed case, epidemiological criteria, and chest imaging). We found 1 (0.30%) “confirmed case”, 2 (0.61%) “probable cases”, 2 (0.61%) “suspected cases”, and 68 (20.73%) with “no sufficient evidence” among the patients’ records coded with ICD-10 code U07.1. We found one (0.30%) “confirmed case” among the patients’ records coded with ICD-10 code U07.2. Additionally, we found one (0.30%) case with “no sufficient evidence” among the patients’ records coded with ICD-10 code B34.2. We found 164 (50%) “confirmed cases” and 18 (5.49%) with “no sufficient evidence” among the patients’ records coded with ICD-10 code B97.2. A total of 60 (18.29%) patients’ records were coded using both ICD-10 codes B97.2 and U07.1, and the majority of them (*n* = 52, 15.85%) were “confirmed cases”. The number of “confirmed cases” with a positive COVID-19 laboratory test was 194 (59.15%). Furthermore, we found 24 (7.32%) “confirmed cases” with no laboratory test but reported from infection control as positive laboratory tests. There were 92 (28.05%) patients’ records with negative laboratory tests, as the following: 83 (25.30%) “no sufficient evidence”, 7 (2.13%) “probable cases”, and 2 (0.61%) “suspected cases”. We found 117 (35.67%) records with documentation on the history of contact with either a probable or a confirmed case, and 90 (27.44%) of these records were “confirmed cases”. Documentation on the epidemiological criteria was generally low and appeared in only 42 (12.80%) patients’ records in our dataset. The majority of “confirmed cases” (*n* = 197, 60.06%) did not include documentation on the epidemiological criteria. Most of the patients’ records that included documentation on the findings of chest imaging were for “confirmed cases” (*n* = 103, 31.40%), followed by patients’ records with “no sufficient evidence” (*n* = 8, 2.44%), and “probable cases” (*n* = 3, 0.91%). Overall, most patients’ records in our dataset were “confirmed cases” (*n* = 218, 66.46%), followed by patients’ records with “no sufficient evidence” (*n* = 91, 27.74%), then “probable cases” (*n* = 10, 3.05%), and lastly “suspected cases” (*n* = 9, 2.74%). Among our dataset, there were 28 (8.54%) death cases reported during admission: 24 (7.32%) “confirmed cases”, 1 (0.30%) “probable case”, 1 (0.30%) “suspected case”, and 2 (0.61%) “no sufficient evidence”.

Table 4 shows the diagnostic accuracy for the identification of patients’ records with “confirmed cases” in the EHR. We found that ICD-10 code B97.2 had the highest sensitivity (99.08%) for the identification of “confirmed cases”. On the other hand, we found that the specificity (100%) for the identification of “confirmed cases” was highest in laboratory tests and ICD-10 code U07.1. We also found that laboratory tests showed the highest accuracy (92.68%) followed by ICD-10 code B97.2 (85.37%). Overall, using the COVID-19 laboratory test to identify “confirmed cases” outperformed the use of ICD-10 codes.

Finally, we found inaccuracy and inconsistency issues between ICD-10 codes and laboratory results. Out of 218 (66.46%) patients’ records who were true “confirmed cases”, we found that 165 (50.30%) cases were not coded using ICD-10 code U07.1. We also found one (0.30%) case was miscoded as ICD-10 code U07.2 even though there was a positive COVID-19 laboratory result. The majority of cases (*n* = 72, 21.95%) were miscoded using ICD-10 code U07.1 even though these cases were not “confirmed cases” (Table 2).

## 4. Discussion

Patients’ data stored in EHR systems are a great source for researchers and experts to use in building predictive modeling systems and real-time public health reporting and surveillance systems. However, EHR data possesses many issues, including documentation inaccuracies and inconsistencies [14,15,42]. In our study, we manually evaluated COVID-19 patients’ records to assess the quality and readiness of EHR data for secondary use in KSUMC, using WHO case definition guidelines for COVID-19, based on COVID-19 codes, COVID-19 laboratory test, history of contact with a probable or confirmed case, clinical and epidemiological criteria, and chest imaging. Most patients’ records in our dataset were “confirmed cases” followed by patients’ records with “no sufficient evidence. Among our dataset, “confirmed cases” were easier to identify using laboratory results, when compared to “probable cases” and “suspected cases” that require using the clinical and epidemiological criteria. We found that the ICD-10 code with the highest percentage among our dataset was ICD-10 code B97.2. Results from comparing the performance of ICD-10 codes versus laboratory tests showed that laboratory tests outperformed ICD-10 codes in the identification of “confirmed cases”.

Our study resulted in identifying several quality issues. First, we found that the percentage of patients’ records with “no sufficient evidence” might indicate a lack of accurate and complete EHR documentation. Second, our dataset also included cases resulting in death, with the majority classified as “confirmed cases”. It is important to mention that cases classified as “death” within our dataset do not necessarily mean that the reason for death was COVID-19 especially with cases that lack positive COVID-19 laboratory tests, which can be challenging [9] to identify through manual review of EHR data. Third, we found that documentation of ICD-10 codes can be inaccurate when validating these codes using laboratory results. Fourth, we found that the rate of documenting COVID-19 signs and symptoms in unstructured clinical notes was higher than structured diagnoses. At the start of the pandemic in Saudi Arabia, 54% of COVID-19 patients were asymptomatic [43]; however, our study showed that asymptomatic cases were not reported in structured data and were only reported in 4.88% in unstructured notes. Furthermore, a review showed that the most common COVID-19 symptoms included fever (98%), cough (76%), dyspnea (55%), myalgia or fatigue (44%), headache (8%), and diarrhea (3%) [44]. Our results showed that these symptoms were more reported in unstructured clinical notes indicating the need for natural language processing (NLP)-assisted approaches to capture these symptoms from EHR. NLP is used to extract clinical information and unstructured features from clinical notes, such as a bag of words, keywords search, and concept extraction, which can be used in building EHR phenotyping algorithms, either rule-based or machine learning techniques. The most popular technique used in NLP is concept extraction from clinical notes, where standardized terminologies can be used [45]. This problem is not unique to our EHR as it has been reported in another study where 40% of diagnoses appeared in notes [15,46]. Fifth, we found some “confirmed cases” without laboratory testing recorded in the EHR but were confirmed by public health reports contained within clinical notes, which were reviewed manually. This creates a burden of identifying COVID-19 “confirmed cases” if a laboratory test was not performed in the same hospital. Sixth, COVID-19 “suspected cases” and “probable cases” were even more challenging to identify within the EHR than “confirmed cases” because “suspected cases” and “probable cases” were, by definition, dependent on symptoms and epidemiological information that were largely found in clinical notes [9], especially when documentation rates of the epidemiological criteria were low in clinical notes among our dataset. These quality issues in the documentation can cause frustration for analysts and researchers when reviewing and analyzing EHR data [13]. Based on the identified data quality issues in our study, we identified certain informatics strategies for using EHR efficiently and to solve these issues (Box 1).

Box 1 **A list of informatics strategies and recommendations to improve for the use of EHR and solving data quality issues.**

- Conduct similar EHR studies across different institutions to fully understand the barriers of high-quality documentation and secondary use of EHR data with the goal to improve the efficiency and quality of EHR data, EHR documentation, and EHR secondary use.
- Avoid using single diagnosis-based phenotyping strategies to define patients, such as diagnosis codes, because it can lead to inaccurate and biased conclusions with negative implications on clinical research and public health surveillance.
- Define the minimum standard content for documentation for EHR at point-of-care within an institution or across different institutions to address the lack of accurate, consistent, and complete EHR data and documentation.
- Develop structured documentation guidelines to document clinical or epidemiological information that is usually documented in unstructured clinical notes.
- Develop natural language processing and automated methods to mine this information from unstructured clinical notes.
- Build an infrastructure for health information exchange across institutions and implement interoperability standards, which have a significant role in establishing shared and aggregated EHR data, standardizing EHR data, and improving EHR data quality to improve the quality and safety of patients’ care.
- Develop automated data quality assessment and validation tools and methods that can be used before EHR applications in conducting secondary research studies, building phenotyping algorithms, and performing data analytics.
- Encourage educational and training efforts to motivate healthcare providers with the importance and benefits of accurate and complete documentation at the point of care.
- Build a multi-disciplinary collaborative team during the initial stages of the clinical crisis could address many of the data quality challenges.

There are some lessons learned and recommendations derived from our real-world EHR data study. First, conducting research studies and deriving causal inferences from EHR data should be carried out with caution as the issues discussed of inaccurate, incomplete, inconsistent, and biased data might arise [9,24]. EHRs might not capture or reflect the patient’s complete health status because patient information can be fragmented across different hospitals or clinics [24]. Furthermore, relying only on structured data is not sufficient and might lead to inaccurate results and conclusions e.g., ICD-10 diagnosis codes. Second, with the current state of EHR systems where information is mostly hidden within unstructured clinical data, we would like to highlight the importance and value of these unstructured textual reports. With manual chart review being cumbersome, expensive, and time-consuming, NLP methods have a crucial role in mining clinical notes, and if adopted, it will lead to a more comprehensive view of the patient. More than 80% of currently available healthcare data are hidden in the unstructured text [47], where there is an underutilization of text. For instance, patient symptoms are not always reported in structured EHR, whereas NLP methods can address this limitation [48], which is also confirmed by our study findings. It is not feasible to capture all information hidden in text using manual methods, especially when dealing with them on a large scale. NLP can be advantageous in identifying patients at risk, building clinical decision support systems, increasing the capacity of healthcare systems, and conducting large-scale studies or population management strategies [49,50]. Third, we believe that the value of accurate clinical documentation might still be underestimated and undervalued by health practitioners. While it is understandable that there are variations in goals between healthcare providers documenting at the point of care and researchers using the data for secondary purposes, it is however important to support the accurate documentation process of both structured and free-text information at the point of care. Downey et al. measured the perceptions of Nurses and Midwives around EHR clinical data quality and found that only 46.3% of them received formal data quality education [29]. By motivating healthcare providers and increasing educational and training efforts, highlighting the benefits of accurate documentation, we may be able to decrease the number of quality-related issues in data [9]. Fourth, there is an increased use of EHR for research purposes and secondary use. Our study showed that the identification of COVID-19 cases (confirmed, probable, susceptible) can be challenging and time-consuming as it requires an extensive amount of manual review. The quality assurance of data and accurate use of standardized terminologies are important components for developing future phenotyping algorithms to identify COVID-19 cases with high performance for secondary-use research [51]. On an international level, lessons learned from the COVID-19 pandemic showed that there is a need to improve international research utilizing clinical data through connecting efforts from multiple countries to expand the capability of dealing with pandemic emergencies worldwide [21]. Fifth, data-driven and AI systems used for disease detection as well as diagnosis and prognostic prediction [52] require high-quality and accurate data. Population health management algorithms that use EHR data to predict or identify patients at risk for a disease, death, or hospitalization to enable providers to identify those patients and engage them to enroll in disease management programs. Such algorithms might be correct, but there may be concerns about data quality that can affect the validity and performance of algorithms [53]. On a national level in Saudi Arabia, for example, the Saudi Data & Artificial Intelligence Authority (SADIA) [54] was established in 2019 to create a data-driven and AI-supported government largely focused on the healthcare sector. Sixth, previous experiences of COVID-19 for leveraging EHR showed that building a multi-disciplinary collaborative team during the early stages of the crisis rather than later could address many of the data and definition challenges, which led to higher-quality data. The collaborative engagement between informaticians, clinicians, data analytics, and researchers as well as team structure re-invention helps to support a cultural shift in handling EHR data at different stages of clinical processes, especially during the pandemic, where accurate, consistent, and high-quality data are required [9,46,55,56]. With these insights and initiatives put in place, ensuring data quality and the application of documentation standards are important facilitators of the advancements of healthcare and translational science.

There were several limitations in our work. Using EHR data alone might limit the generalizability of our findings, where there might be variations across EHR systems or within the same hospital system over time [10,24]. Even though we identified challenges and issues within a single EHR system, these challenges and issues might not be unique to a single EHR and can exist anywhere [13]. In addition, the EHR system used in our institution is a vendor-based system that is widely used. Future work should focus on comparative studies to improve our understanding of potential variations across different EHRs on a national level. Quality assessment in our study was performed manually utilizing WHO guidelines for COVID-19; however, it was a time-consuming, cumbersome, and non-scalable process. For application to a larger population and more phenotypes, there is a need to build automated quality assessment tools that can be used to validate EHR data before its use. Finally, we encourage the exploration of documentation challenges among health workers and their perspectives about the EHR documentation interface.

## 5. Conclusions

More attention should be given to data quality and limitations of EHR. This study demonstrates the existing shortcomings in the documentation where data quality evaluation should be incorporated when utilizing EHR data to ensure patient safety during documentation and to ensure data readiness for secondary use and future applications of research and predictive models. We chose to evaluate COVID-19 data quality to provide an example of potential limitations that might be faced using EHR data when conducting COVID-19-related research using real-world data. We used real-world patient-level data, which usually might not be available for every researcher. Documentation rates of diagnoses were lower in structured diagnoses than in unstructured clinical notes. Using laboratory results for COVID-19 case identification is more accurate than ICD-10 codes as ICD-10 codes do not necessarily reflect the patient’s accurate health status. We encourage educational and training efforts to motivate healthcare providers with the importance and benefits of accurate and complete documentation at the point-of-care. Furthermore, building a multi-disciplinary collaborative team as well as data analytics during the initial stages of the clinical crisis could address many of the quality data challenges. Finally, future research should focus on building automated quality assessment tools that can be used prior to EHR applications in conducting secondary research studies, building phenotyping algorithms, and performing data analytics.

## Figures and Tables

**Table 1:** COVID-19 Cases: Inclusionary ICD codes.

ICD-10 Codes	Code Description
U07.1	COVID-19, virus identified. The code is assigned to a disease diagnosis of COVID-19 confirmed by laboratory testing.
U07.2	COVID-19, virus not identified. The code is assigned to a clinical or epidemiological diagnosis of COVID-19 where laboratory confirmation is inconclusive or not available.
B34.2	Coronavirus infection, unspecified site.
B97.2	Coronavirus as the cause of 0020 diseases classified to other chapters.

**Table 2:** Descriptive summary of COVID-19 dataset.

Characteristic	Frequency	%
**Gender**		
Female	139	42.38%
Male	189	57.62%
**Age (Years)**		
Less than or equal to 10	15	4.57%
11–20	18	5.49%
21–30	39	11.89%
31–40	69	21.04%
41–50	38	11.59%
51–60	57	17.38%
61–70	50	15.24%
71+	42	12.80%
**Nationality**		
Saudi	233	71.04%
Non-Saudi	95	28.96%
**Medical departments (by encounters)**		
Medical (General, Cardiology, Endocrinology, Gastroenterology, Hematology, Nephrology, Neurology, Oncology, Pulmonary, Rheumatology)	258	71.47%
Gynecology-Obstetrics	46	12.74%
Surgery (General, Neurosurgery, Orthopedics, Plastic, Peripheral Vascular, Pediatric, Urology)	24	6.65%
Emergency Medicine	16	4.43%
Pediatric (General, Hematology, Infectious Disease, Neonatology, Nephrology)	15	4.16%
Ophthalmology	1	0.28%
Ear, nose, and throat (ENT)	1	0.28%

**Table 3:** Patients’ records description linked to final interpretations.

Item	Confirmed Case (% of 328)	Probable Case (% of 328)	Suspected Case (% of 328)	No Sufficient Evidence (% of 328)
**COVID-19 ICD-10 codes**				
U07.1	1 (0.30%)	2 (0.61%)	2 (0.61%)	68 (20.73%)
U07.2	1 (0.30%)	0 (0%)	0 (0%)	0 (0%)
B34.2	0 (0%)	0 (0%)	0 (0%)	1 (0.30%)
B97.2	164 (50%)	7 (2.13%)	4 (1.22%)	18 (5.49%)
U07.1 and B97.2	52 (15.85%)	1 (0.30%)	3 (0.91%)	4 (1.22%)
**COVID-19 Laboratory test**				
Positive	194 (59.15%)	0 (0%)	0 (0%)	0 (0%)
Positive (results obtained from infection and control report within patients’ records)	24 (7.32%)	0 (0%)	0 (0%)	0 (0%)
Negative	0 (0%)	7 (2.13%)	2 (0.61%)	83 (25.30%)
No laboratory test found	0 (0%)	3 (0.91%)	7 (2.13%)	8 (2.44%)
**History of Contact with a probable or confirmed case**				
Yes	90 (27.44%)	8 (2.44%)	2 (0.61%)	17 (5.18%)
No	128 (39.02%)	2 (0.61%)	7 (2.13%)	74 (22.56%)
**Epidemiological criteria**				
(1) Residing or working in a setting with high risk of transmission of the virus	14 (4.27%)	0 (0%)	7 (2.13%)	5 (1.52%)
(2) Working in a health setting, including within health facilities and within households.	4 (1.22%)	1 (0.30%)	2 (0.61%)	2 (0.61%)
(3) Residing in or travel to an area with community transmission anytime (e.g., China, Iran)	3 (0.91%)	0 (0%)	0 (0%)	3 (0.91%)
(1) and (2)	0 (0%)	0 (0%)	0 (0%)	1 (0.30%)
None (No information is documented about epidemiological criteria)	197 (60.06%)	9 (2.74%)	0 (0%)	80 (24.39%)
**Chest Imaging**				
Evidence of COVID-19	103 (31.40%)	3 (0.91%)	0 (0%)	8 (2.44%)
No evidence of COVID-19	66 (20.12%)	5 (1.52%)	8 (2.44%)	31 (9.45%)
No chest imaging was found	49 (14.94%)	2 (0.61%)	1 (0.30%)	52 (15.85%)
**Total**	218 (66.46%)	10 (3.05%)	9 (2.74%)	91 (27.74%)

**Table 4:** The frequencies of using ICD-10 codes and the diagnostic accuracy between ICD-10 codes and laboratory tests for identification of confirmed COVID-19 cases.

Item	Number of Records (% of 328)	Sensitivity	Specificity	Accuracy
**COVID-19 ICD-10 codes**				
U07.1	133 (40.55%)	24.31%	27.27%	25.30%
U07.2	1 (0.30%)	0.46%	100%	33.84%
B34.2	1 (0.30%)	0%	99.09%	33.23%
B97.2	253 (77.13%)	99.08%	66.36%	85.37%
U07.1 and B97.2	60 (18.29%)	23.85%	92.73%	46.95%
**COVID-19 Laboratory test**				
Positive	194 (59.15%)	89%	100%	92.68%

## Data Availability

Not applicable.

## Appendix A

**Table A1:** The Structured Form Was Used for Chart Review.

▪ Clinical Criteria (Please select all symptoms or signs within 10 days) appeared in the list of diagnoses and problems (Check under Diagnosis list) * ▯ Fever ▯ Fever of ≥38 °C ▯ Cough ▯ Severe acute respiratory illness ▯ General weakness/fatigue ▯ Headache ▯ Myalgia ▯ Sore throat ▯ Coryza ▯ Dyspnoea ▯ Anorexia/nausea/vomiting ▯ Diarrhea ▯ Altered mental status ▯ No symptoms or signs were found ▯ Asymptomatic ▯ Anosmia (loss of smell) ▯ Ageusia (loss of taste) ▯ None found ▪ Clinical Criteria (Please select all symptoms or signs) appeared in clinical notes (Check notes in E-Sihi) * ▯ Fever (not specified or less than 38 °C) ▯ Fever of ≥38 °C ▯ Cough ▯ Severe acute respiratory illness ▯ General weakness/fatigue ▯ Headache ▯ Myalgia ▯ Sore throat ▯ Coryza ▯ Dyspnoea ▯ Anorexia/nausea/vomiting ▯ Diarrhea ▯ Altered mental status ▯ No symptoms or signs were found ▯ Asymptomatic ▯ Anosmia (loss of smell) ▯ Ageusia (loss of taste) ▯ None found ▪ How many notes did you review for this patient? ▪ Epidemiological criteria (Please select all applicable) (Check notes in E-Sihi) * ▯ Residing or working in a setting with a high risk of transmission of the virus ▯ Residing in or travel to an area with community transmission anytime within the 14 days before symptom onset (e.g., China, Iran) ▯ Working in a health setting, including within health facilities and within households, anytime within the 14 days before symptom onset. ▯ In contact of a probable or confirmed case within the previous 10–14 days ▯ None (No information is documented about epidemiological criteria) ▪ Laboratory test (COVID-19 or COVID-19 Drive-Thru)—check tests or infection control (Check under Labs) * ▯ Positive ▯ Negative ▯ No laboratory test found ▯ No laboratory test was found, but a Report from infection control for a positive laboratory test. ▪ Chest imaging report showing findings suggestive of COVID-19 disease: (Check under imaging in E-Sihi) * ▯ Yes ▯ No ▯ No chest imaging ▪ Your final interpretations (based on WHO guidelines): The SARS-CoV-2 infection final diagnosis based on EHR data review is * ▯ Suspected case ▯ Probable case ▯ Confirmed case ▯ No sufficient evidence

**Table A2:** Diagnosis Criteria Used in Chart Review and Data Analysis.

Laboratory Criteria	Positive Nucleic Acid Amplification Test (NAAT)
**Clinical criteria**	A person who has three or more of the following symptoms: fever, cough, general weakness/fatigue, 1 headache, myalgia, sore throat, coryza, dyspnoea, anorexia/nausea/vomiting, diarrhea, altered mental status. A person who has one or more of the following symptoms: shortness of breath, cough, or difficulty breathing. A Person with Severe respiratory illness with one or more of the following: Pneumonia confirmed clinically or radiologically, OR Acute respiratory distress syndrome (ARDS) with no other diagnosis.
**Epidemiologic Criteria (within the 14 days before symptom onset)**	Residing or working in a setting with a high risk of transmission of the virus; OR Working in a health setting, including within health facilities and within households; OR Residing in or travel to an area with community transmission.
**Chest Imaging**	Findings suggestive of COVID-19.

**Table A3:** Signs and Symptoms in Both Structured and Unstructured Data.

Signs and Symptoms	Structured	% of 328	Unstructured	% of 328
Ageusia (loss of taste)	0	0%	5	1.52%
Altered mental status	1	0.30%	5	1.52%
Anorexia/nausea/vomiting	14	4.27%	62	18.90%
Anosmia (loss of smell)	0	0%	8	2.44%
Asymptomatic	0	0%	16	4.88%
Coryza	46	14.02%	30	9.15%
Cough	39	11.89%	139	42.38%
Diarrhea	3	0.91%	39	11.89%
Dyspnea	97	29.57%	140	42.68%
Fever	74	22.56%	151	46.04%
General weakness/fatigue	4	1.22%	20	6.10%
Headache	18	5.49%	32	9.76%
Myalgia	4	1.22%	24	7.32%
Severe acute respiratory illness	10	3.05%	14	4.27%
Sore throat	13	3.96%	40	12.20%
No symptoms or signs were found	129	39.33%	85	25.91%
